# Novel Unsymmetrical Ru(III) and Mixed-valence Ru(III)/Ru(II) Dinuclear Compounds Related to the Antimetastatic Ru(III) Drug NAMI-A

**DOI:** 10.1155/MBD.2001.9

**Published:** 2001

**Authors:** B. Serli, E. Iengo, T. Gianferrara, E. Zangrando, E. Alessio

**Affiliations:** 1 Dipartimento di Scienze Chimiche University of Trieste Via L. Giorgieri 1 Trieste 34127 Italy; 2 Dipartimento di Scienze Farmaceutiche University of Trieste Piazzale Europa 1 Trieste 34127 Italy

## Abstract

In this paper we report the stepwise preparation and the characterization of new unsymmetrical monoanionic Ru(III) dinuclear compounds, [NH_4_][{*trans*-RuCl_4_(Me_2_SO-S)}(μ-L){*mer*-RuCl_3_(Me_2_SO-S)(Me_2_SO-O)}] (L = pyz (**1**), pym (**2**)). By a similar synthetic approach we also prepared new mixed-valence Ru(III)/Ru(II) dinuclear compounds of formula [NH_4_][{*trans*-RuCl_4_(Me_2_SO-S)}(μ-pyz){*cis,cis,cis*-RuCl_2_(Me_2_SO-S)_2_(CO)}] (L = pyrazine (pyz, **3**), pyrimidine (pym, **4**)). Moreover, we describe the chemical behavior of compounds **1-4** in physiological solution, also after complete reduction (with ascorbic acid) to the corresponding Ru(II)/Ru(II) species. Overall, the chemical behavior of **1** and **2** after reduction resembles that of the corresponding dianionic and neutral dinuclear species, [{*trans*-RuCl_3_(Me_2_SO-S)}_2_(μ-L)]^2−^and [{*mer*-RuCl_3_(Me_2_SO-S)(Me_2_SO-O)}_2_ (μ-L)]. On the other hand, the mixed-valence dinuclear compounds **3** and **4**, owing to the great inertness of the cis,cis,cis-RuCl_2_(Me_2_SO-S)_2_(CO)(1/2μ-L) fragment, behave substantially like the mononuclear species [*trans*-RuCl_4_(Me_2_SO-S)(L)]^−^ in which the terminally bonded L ligand can be considered as bearing a bulky substituent on the other N atom.


### NOVEL UNSYMMETRICAL Ru(III) AND MIXED-VALENCE Ru(III)/Ru(II) DINUCLEAR COMPOUNDS RELATED TO THE ANTIMETASTATIC Ru(III) DRUG NAMI-A

B. Serli,[1] E. Iengo,[1] T. Gianferrara,[2] E. Zangrando,[1] and E. Alessio[1]*

[1] Dipartimento di Scienze Chimiche, University of Trieste, Via L. Giorgieri 1, 34127 Trieste, Italy
[2] Dipartimento di Scienze Farmaceutiche, University of Trieste, Piazzale Europa 1, 34127 Trieste, Italy

### ABSTRACT

In this paper we report the stepwise preparation and the characterization of new unsymmetrical monoanionic Ru(III) dinuclear compounds, $[NH_4][\{trans\text{-}RuCl_4(Me_2SO\text{-}S)\}(\mu\text{-}L)\{mer\text{-}RuCl_3(Me_2SO\text{-}S)(Me_2SO\text{-}O)\}]$ (L = pyz (**1**), pym (**2**)). By a similar synthetic approach we also prepared new mixed-valence Ru(III)/Ru(II) dinuclear compounds of formula $[NH_4][\{trans\text{-}RuCl_4(Me_2SO\text{-}S)\}(\mu\text{-}pyz)\{cis,cis,cis\text{-}RuCl_2(Me_2SO\text{-}S)_2(CO)\}]$ (L = pyrazine (pyz, **3**), pyrimidine (pym, **4**)). Moreover, we describe the chemical behavior of compounds **1-4** in physiological solution, also after complete reduction (with ascorbic acid) to the corresponding Ru(II)/Ru(II) species. Overall, the chemical behavior of **1** and **2** after reduction resembles that of the corresponding dianionic and neutral dinuclear species, $[\{trans\text{-}RuCl_4(Me_2SO\text{-}S)\}_2(\mu\text{-}L)]^{2-}$ and $[\{mer\text{-}RuCl_3(Me_2SO\text{-}S)(Me_2SO\text{-}O)\}_2(\mu\text{-}L)]$. On the other hand, the mixed-valence dinuclear compounds **3** and **4**, owing to the great inertness of the $cis,cis,cis\text{-}RuCl_2(Me_2SO\text{-}S)_2(CO)(1/2\mu\text{-}L)$ fragment, behave substantially like the mononuclear species $[trans\text{-}RuCl_4(Me_2SO\text{-}S)(L)]^-$ in which the terminally bonded L ligand can be considered as bearing a bulky substituent on the other N atom.

### INTRODUCTION

The introduction of the Ru(III)-dimethylsulfoxide complex $ImH[trans\text{-}RuCl_4(Me_2SO\text{-}S)Im]$ (NAMI-A, Im = imidazole, [1]) into phase I clinical trials as antimetastatic drug (October 1999) prompted us to investigate also other closely related ruthenium compounds. Within this frame, stimulated by the excellent results obtained by the platinum anticancer dimers and trimers developed by Farrell [2], we prepared also symmetrical dinuclear ruthenium(III) species, either dianionic $[Na]_2[\{trans\text{-}RuCl_4(Me_2SO\text{-}S)\}_2(\mu\text{-}L)]$ (**a**), or neutral $[\{mer\text{-}RuCl_3(Me_2SO\text{-}S)(Me_2SO\text{-}O)\}_2(\mu\text{-}L)]$ (**b**), with L = bridging heterocyclic N-donor ligand such as pyrazine (pyz), pyrimidine (pym), 4,4'-bipyridine (bipy) and derivatives thereof (Figure 1) [3].

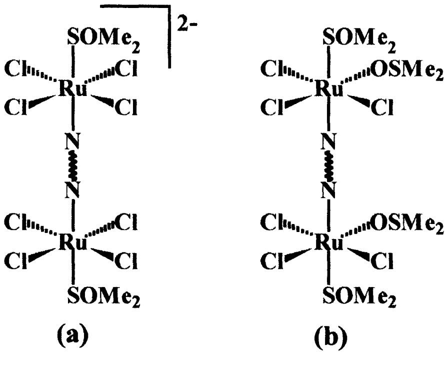

Figure 1. Schematic representation of the dianionic (**a**) and neutral (**b**) symmetrical dimers described in ref 3.

The coordination environment of each ruthenium(III) center in the dianionic species **a** is essentially the same as in the mononuclear compound NAMI-A (four trans chlorides, one S-bonded dimethylsulfoxide and one heterocyclic N-ligand). Preliminary results from *in vivo* tests on the murine MCa carcinoma model showed that some of the dianionic species were as effective as NAMI-A in reducing the spontaneous metastases (to about 5% with respect to controls) at a dosage 3.5 times lower in terms of ruthenium compared to that normally used for NAMI-A [4]. The neutral dinuclear compounds **b**, based on the known chemical behavior of the corresponding mononuclear species $mer\text{-}RuCl_3(Me_2SO\text{-}S)(Me_2SO\text{-}O)(L)$ [5], are expected to behave differently compared to the dianionic analogs **a** and might therefore give different biological responses as well; however, they could not be tested owing to very low solubility in aqueous solution.

We thought thus of combining the chemical environments of both neutral and dianionic species and prepared a new series of unsymmetrical dinuclear compounds, $[NH_4]$[{*trans*-$RuCl_4(Me_2SO$-*S*)}(μ-L){*mer*-$RuCl_3(Me_2SO$-*S*)$(Me_2SO$-*O*)}] (L = pyz (**1**), pym (**2**), Figure 2), whose negative charge is expected to provide appreciable solubility in aqueous solution. By a similar synthetic approach we also prepared new mixed-valence Ru(III)/Ru(II) dinuclear compounds of formula $[NH_4]$[{*trans*-$RuCl_4(Me_2SO$-*S*)}(μ-pyz){*cis,cis,cis*-$RuCl_2(Me_2SO$-*S*$)_2$(CO)}] (L = pyz (**3**), pym (**4**), Figure 2).

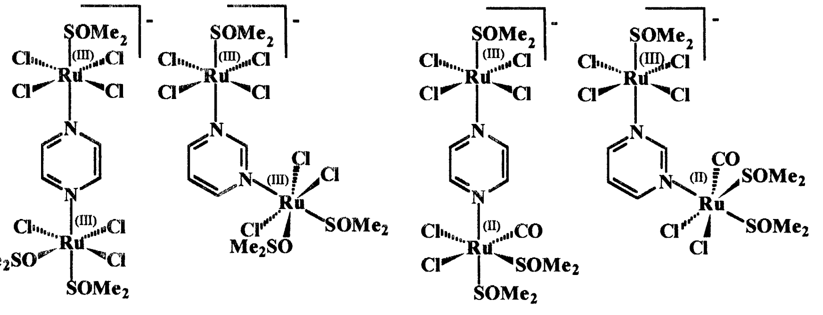

1 2 3 4

Figure 2. Schematic representation of the unsymmetrical dinuclear compounds **1**-**4**.

Beside the preparation and characterization of the novel unsymmetrical dinuclear compounds **1**-**4**, in this paper we also describe their chemical behavior in physiological solution and after complete reduction (with ascorbic acid) to the corresponding Ru(II)/Ru(II) species.

### MATERIALS AND METHODS

The bridging ligands were purchased from Aldrich and used as received. Electronic absorption spectra were recorded in quartz cells with a Jasco UV/vis V500 spectrophotometer equipped with a Peltier thermostatic unit. Infrared spectra were obtained on a Perkin-Elmer 983 G spectrometer. $^1$H-NMR spectra ($D_2O$, Aldrich) were collected at 400 MHz on a Jeol EX 400 FT spectrometer; spectra were recorded at room temperature with 2,2-dimethyl-2,2-silapentane-5-sulfonate (DSS) as an internal standard. An inversion recovery pulse sequence ($\pi$ - $\tau$ - $\pi/2$) was applied for recording the spectra of the paramagnetic compounds (spectral window 30.000 Hz, pulse delay 0.5 s); with $\tau$ = 100 ms the resonances of molecules not coordinated to Ru(III) were negative, while those of the coordinated ligands were positive. A 0.1 mol $dm^{-3}$ pH* 7.4 phosphate-$D_2O$ buffer (pH meter reading from $D_2O$ solutions) containing 0.9% NaCl was used for NMR experiments aimed to monitor the chemical behavior of the dimers in physiological solution.

The complexes $[(Me_2SO)_2H]$[*trans*-$RuCl_4(Me_2SO$-*S*$)_2$] [6], *mer,cis*-$RuCl_3(Me_2SO$-*S*)$(Me_2SO$-*O*)(pyz) [3], *mer,cis*-$RuCl_3(Me_2SO$-*S*)$(Me_2SO$-*O*)(pym) [3], *cis,fac*-$RuCl_2(Me_2SO)_3$(CO) [7], *cis,cis,cis*-$RuCl_2(Me_2SO$-*S*$)_2$(CO)(pyz) [7], and *cis,cis,cis*-$RuCl_2(Me_2SO$-*S*$)_2$(CO)(pym) [7] were prepared according to the reported procedures.

**Preparation of $[NH_4]$[*trans*-$RuCl_4(Me_2SO$-*S*$)_2$].** A 0.096 g amount of $NH_4Cl$ (1.80 mmol) dissolved in 0.3 mL of water was added under stirring to a clear orange-red solution of $[(Me_2SO)_2H]$[*trans*-$RuCl_4(Me_2SO$-*S*$)_2$] (0.50 g, 0.90 mmol) in aqueous ethanol (0.3 mL of $H_2O$ and 22.5 mL of ethanol). A deep-orange precipitate formed in a few minutes; after standing overnight at 4 °C, it was collected by filtration, washed with cold acetone, and diethyl ether, and vacuum dried at ambient T (0.29 g, 78%). Found C, 11.4; H, 3.82; N, 3.23%. $C_4H_{16}NCl_4O_2S_2Ru$ requires C, 11.5; H, 3.86; N, 3.35%. UV/vis ($\lambda_{max}$, nm) in $H_2O$: 398, 465. $^1$H NMR ($D_2O$, ppm, 400 MHz): -16.7 (br, $Me_2SO$-*S*).

**Preparation of $[NH_4]$[{*trans*-$RuCl_4(Me_2SO$-*S*)}(μ-pyz){*mer*-$RuCl_3(Me_2SO$-*S*)$(Me_2SO$-*O*)}] (1).** A 0.053 g amount of *mer,cis*-$RuCl_3(Me_2SO$-*S*)$(Me_2SO$-*O*)(pyz) (0.12 mmol) dissolved in 7 mL of $CHCl_3$ was added to a methanol solution (7 mL) of $[NH_4]$[*trans*-$RuCl_4(Me_2SO$-*S*$)_2$] (0.063 g, 0.15 mmol). A red crystalline precipitate formed within two days at ambient temperature, and was then collected by filtration, washed with cold methanol and diethyl ether, and vacuum dried at ambient T (0.044 g, 47%). According to elemental analysis and NMR spectroscopy the product contains one $CH_3OH$ molecule of crystallization; found C, 16.1; H, 3.60; N, 5.04%. $C_{11}H_{30}N_3Cl_7O_4S_3Ru_2$ requires C, 16.2; H, 3.71; N, 5.15%. UV/vis ($\lambda_{max}$, nm) in $H_2O$: 381. $^1$H NMR ($D_2O$, ppm, 400 MHz): 9.9 (br, $Me_2SO$-*O*), -14.2 (br, $Me_2SO$-*S*). IR (KBr, $cm^{-1}$): ν(S-O) 1111 (vs, $Me_2SO$-*S*), 906 (vs, $Me_2SO$-*O*); ν(Ru-O) 519 (m); ν(Ru-S) 426 (m); ν(Ru-Cl) 338 (vs).

**Preparation of [NH$_4$][{*trans*-RuCl$_4$(Me$_2$SO-*S*)}(μ-pym){*mer*-RuCl$_3$(Me$_2$SO-*S*)(Me$_2$SO-*O*)}] (2).** The complex was prepared by the method used for **1** (yield 37%). According to elemental analysis and NMR spectroscopy the product contains one CH$_3$OH molecule of crystallization; found C, 16.0; H, 3.61; N, 5.07%. C$_{11}$H$_{30}$N$_3$Cl$_7$O$_4$S$_3$Ru$_2$ requires C, 16.2; H, 3.71; N, 5.15%. UV/vis ($\lambda_{max}$, nm) in H$_2$O: 385. $^1$H NMR (D$_2$O, ppm, 400 MHz): 11.4 (br, Me$_2$SO-*O*), -9.1 (br, pym), -14.4 (br, Me$_2$SO-*S*). IR (KBr, cm$^{-1}$): ν(S-O) 1093 (vs, Me$_2$SO-*S*), 906 (vs, Me$_2$SO-*O*); ν(Ru-O) 501 (m); ν(Ru-S) 428 (m); ν(Ru-Cl) 335 (vs).

**Preparation of [NH$_4$][{*trans*-RuCl$_4$(Me$_2$SO-*S*)}(μ-pyz){*cis,cis,cis*-RuCl$_2$(Me$_2$SO-*S*)$_2$(CO)}] (3).** A 0.05 g amount of *cis,cis,cis*-RuCl$_2$(Me$_2$SO-*S*)$_2$(CO)(pyz) (0.115 mmol) dissolved in 7 mL of CHCl$_3$ was added to a methanol solution (5.5 mL) of [NH$_4$][*trans*-RuCl$_4$(Me$_2$SO-*S*)$_2$] (0.054 g, 0.13 mmol). An orange precipitate formed within two days at 4 °C and was then collected by filtration, washed with cold methanol and diethyl ether, and vacuum dried at ambient T (0.032 g, 35%). According to elemental analysis the product contains one H$_2$O molecule of crystallization; found C, 16.2; H, 3.25; N, 5.07%. C$_{11}$H$_{28}$N$_3$Cl$_6$O$_5$S$_3$Ru$_2$ requires C, 16.3; H, 3.45; N, 5.18%. UV/vis ($\lambda_{max}$, nm) in H$_2$O: 471, 402. $^1$H NMR (D$_2$O, ppm, 400 MHz): 2.82, 2.73, 2.45, 2.13 (s, Me$_2$SO-*S*–Ru(II)), -0.4 (br, pyz), -13.8 (br, Me$_2$SO-*S*–Ru(III)). IR (KBr, cm$^{-1}$): ν(CO) 2007 (vs); ν(S-O) 1123 (vs, Me$_2$SO-*S*); ν(Ru-S) 429 (m).

**Preparation of [NH$_4$][{*trans*-RuCl$_4$(Me$_2$SO-*S*)}(μ-pym){*cis,cis,cis*-RuCl$_2$(Me$_2$SO-*S*)$_2$(CO)}] (4).** The complex was prepared by the method used for **3** (yield 34%). Found C, 17.0; H, 3.35; N, 5.30%. C$_{11}$H$_{26}$N$_3$Cl$_6$O$_4$S$_3$Ru$_2$ requires C, 17.0; H, 3.38; N, 5.42%. UV/vis ($\lambda_{max}$, nm) in H$_2$O: 471, 402. $^1$H NMR (D$_2$O, ppm, 400 MHz): 3.26, 3.11 (s, Me$_2$SO-*S*–Ru(II)), 7.0, 3.4, -1.0 (br, pym), -14.0 (br, Me$_2$SO-*S*–Ru(III)). IR (KBr, cm$^{-1}$): ν(CO) 2007 (vs); ν(S-O) 1123 (vs, Me$_2$SO-*S*); ν(Ru-S) 429 (m).

**Crystallography**

**Crystal data for [NH$_4$][{*trans*-RuCl$_4$(Me$_2$SO-*S*)}(μ-pyz){*mer*-RuCl$_3$(Me$_2$SO-*S*)(Me$_2$SO-*O*)}]·CH$_3$OH (1)**

Empirical formula, C$_{11}$H$_{30}$Cl$_7$N$_3$O$_4$Ru$_2$S$_3$. M = 814.85; System, *triclinic*; Space group, *P*-1; *a* = 8.523(1); *b* = 12.173(4); *c* = 14.553(4) Å; α = 90.97(2); β = 95.44(1); γ = 107.94(2)°; Vol. = 1428.3(6) Å$^3$; *Z* = 2; $D_{calc}$ = 1.895 g cm$^{-3}$; $\mu$ (Mo-*K*α) = 1.954 mm$^{-1}$; F(000) 808; of 6858 measured data (room temperature, $2\theta_{max}$ = 56°), 6588 were unique (Rint = 0.0278), 4818 observed [$I > 2\sigma(I)$]; 282 parameters; $R1(F^2)$ = 0.0509; $wR2(F^2)$ = 0.1724; ΔF max, min: 1.473, -1.492 eÅ$^{-3}$. Deposition number CCDC 156559.

**Structure determination**

The dataset was collected at room temperature on an Enraf-Nonius CAD4 diffractometer, equipped with graphite monochromator and Mo-*K*α radiation (λ = 0.71073 Å). Intensities data were corrected for Lorentz-polarization effects and absorption through an empirical ψ-scan method. The structure was solved by conventional Patterson and Fourier technique [8] and refined by full-matrix anisotropic least-squares method [9]. A molecule of methanol was located on the Δ*F* map with the oxygen atom disordered over two positions refined to occupancies of 0.76 and 0.24. All the calculations were performed using the WinGX System, Ver 1.63 [10].

### RESULTS AND DISCUSSION

**Preparation of unsymmetrical monoanionic Ru(III) dinuclear compounds.**

The synthesis of unsymmetrical monoanionic Ru(III) dinuclear species of formula [{*trans*-RuCl$_4$(Me$_2$SO-*S*)}(μ-L){*mer*-RuCl$_3$(Me$_2$SO-*S*)(Me$_2$SO-*O*)}]$^-$ requires a stepwise reaction in which the bridging ligand L is bound first to one Ru(III)-Me$_2$SO precursor, either anionic [*trans*-RuCl$_4$(Me$_2$SO-*S*)$_2$]$^-$, or neutral *mer*-RuCl$_3$(Me$_2$SO)$_3$, and the resulting complex is then treated with the complementary fragment (Scheme 1).

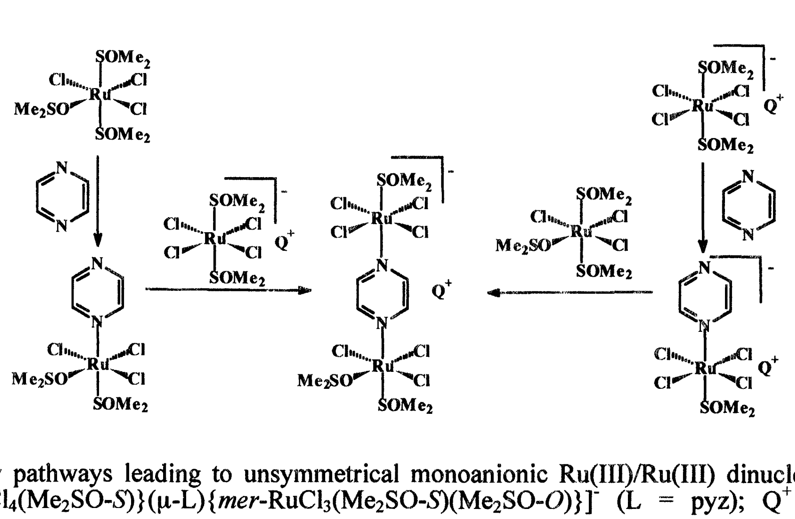

Scheme 1. Reactivity pathways leading to unsymmetrical monoanionic Ru(III)/Ru(III) dinuclear species of formula [{*trans*-RuCl$_4$(Me$_2$SO-*S*)}(μ-L){*mer*-RuCl$_3$(Me$_2$SO-*S*)(Me$_2$SO-*O*)}]$^-$ (L = pyz; Q$^+$ represents a generic cation.

Regardlesss of the pathway chosen, in the crucial step an anionic Ru(III) species has to react with a neutral species and the choice of the most appropriate solvent is of paramount importance. We focused on pyz and pym ligands and found that their coordination to the neutral precursor *mer*-$RuCl_3(Me_2SO)_3$, followed by treatment with the anionic fragment [*trans*-$RuCl_4(Me_2SO$-*S*$)_2]^-$, gives best results. Firstly we found that treatment of [$NEt_4$][*trans*-$RuCl_4(Me_2SO$-*S*$)_2$] with *mer,cis*-$RuCl_3(Me_2SO$-*S*)($Me_2SO$-*O*)(pyz) in chloroform leads to [$NEt_4$][{*trans*-$RuCl_4(Me_2SO$-*S*)}(μ-pyz){*mer*-$RuCl_3(Me_2SO$-*S*)($Me_2SO$-*O*}] in good yield; however this species is unsoluble in water and thus not suited for our purposes. After screening other cations and solvents, we found that the best results were obtained with the ammonium salt [$NH_4$][*trans*-$RuCl_4(Me_2SO$-*S*$)_2$] in methanol/chloroform mixtures. A proper choice of the methanol/chloroform ratio allowed us to obtain the unsymmetrical dinuclear species [$NH_4$][{*trans*-$RuCl_4(Me_2SO$-*S*)}(μ-pyz){*mer*-$RuCl_3(Me_2SO$-*S*)($Me_2SO$-*O*)}] (**1**) in pure form even though with relatively low yield; larger amounts of chloroform induced the co-precipitation of the corresponding dianionic symmetrical dimer $[NH_4]_2$[{*trans*-$RuCl_4(Me_2SO$-*S*)$\}_2$(μ-pyz)], while larger amounts of methanol favored the co-precipitation of the neutral dimer [{*mer*-$RuCl_3(Me_2SO$-*S*)($Me_2SO$-*O*)$\}_2$(μ-pyz)].

Complex **1** is fairly soluble in aqueous solution and was fully characterized by elemental analysis and spectroscopic techniques; the $^1H$ NMR spectrum of **1** ($D_2O$) shows the typical broad resonances at about $\delta$ = -14 and 10 for S-bonded and O-bonded dimethylsulfoxide, respectively, while the resonances of the bridging pyrazine protons are broadened beyond detection by the two close paramagnetic Ru(III) centers. The NMR spectrum of the corresponding dinuclear species with pyrimidine, **2**, shows also a rather broad and weak resonance at $\delta$ = -9 attributed to H5, i.e. the proton of the bridging pym ligand which is further removed from the Ru(III) nuclei. The X-ray structure of **1** was also determined.

**Crystal structure of [$NH_4$][{*trans*-$RuCl_4(Me_2SO$-*S*)}(μ-pyz){*mer*-$RuCl_3(Me_2SO$-*S*)($Me_2SO$-*O*)}] ·$CH_3OH$ (1).**

The crystal of **1** is built up by complex and ammonium ions, and disordered molecules of methanol. Both the Ru(III) ions, linked by the pyrazine ligand, display a pseudo-octahedral coordination (Figure 3). The four chlorides at Ru(1) result almost eclipsed with the chlorides and the oxygen donors at Ru(2), with the pyrazine oriented in such a way to bisect approximately one Cl–Ru–Cl bond angle. The Ru–Cl bond lengths fall in a range from 2.328(3) to 2.377(2) Å.

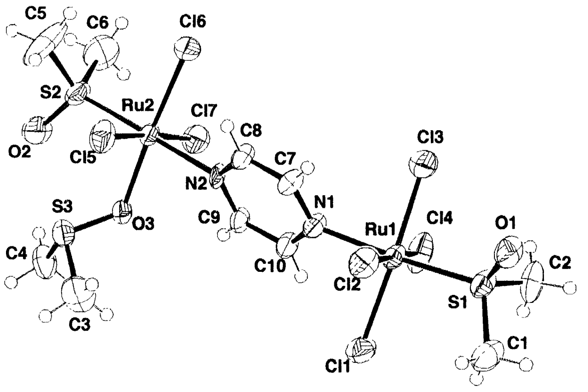

Figure 3. Perpective view of the dinuclear anion of [$NH_4$][{*trans*-$RuCl_4(Me_2SO$-*S*)}(μ-pyz){*mer*-$RuCl_3(Me_2SO$-*S*)($Me_2SO$-*O*)}] (**1**) (thermal ellipsoids at 50% probability level). Relevant bond distances (Å) and angles (°): Ru(1)–N(1) 2.102(6), Ru(1)–S(1) 2.291(2), Ru(2)–N(2) 2.107(6), Ru(2)–S(2) 2.273(2), Ru(2)–O(3) 2.048(5), Ru–Cl range from 2.328(3) to 2.377(2); N(1)–Ru(1)–S(1) 175.1(2), Cl(4)–Ru(1)–Cl(2) 177.54(9), Cl(3)–Ru(1)–Cl(1) 177.65(8), N(2)–Ru(2)–S(2) 177.4(2), O(3)–Ru(2)–Cl(6) 175.8(2), Cl(7)–Ru(2)–Cl(5) 175.62(9).

The arrangements of the $Me_2SO$-*S* ligands are different, and the orientation might account for the difference of about 0.02 Å observed in the Ru–S bond distances (Ru(2)–S(2) 2.273(2) Å, Ru(1)–S(1) 2.291(2) Å) [11]. In fact, in one case the conformation is that usually found in Ru–($Me_2SO$-*S*) complexes [12], with the O–S bond nearly eclipsed with the equatorial Ru(2)–O(3) bond. The torsion angles O(3)–Ru(2)–S(2)–O(2) and Cl(3)–Ru(1)–S(1)–O(1) are 2.6(5) and 42.1(4)°, respectively. The S-coordinated ligands, like the free sulfoxides, are able to give rise to H-bonding with protic solvents [13]. In the present case, the sulfoxide oxygen O(1) interacts electrostatically with two ammonium ions at 2.93 and 2.96 Å, leading to a lengthening of the S(1)–O(1) bond distance (1.489(6) Å), with respect to the value of 1.451(7) Å

observed for S(2)–O(2). These interactions might be also responsible for the elongation detected in the Ru–S(1) bond length. As for the conformation of the $Me_2SO$-*O*, the torsion angles Ru–O–S–C of about $\pm 128°$ indicate a *trans-trans* orientation of the methyl groups, which correponds to a minimum in the strain energy profile [12]. The structure of the anion results slightly bowed along the Ru–pyrazine–Ru vector, as evidenced by the dihedral angles made by the best fit planes through the N base (r.m.s. 0.013) and the Ru(1) and Ru(2) equatorial mean planes of 81.4(2) and 87.8(2)°, respectively, the former being considerably apart from the ideal value of 90°.

Overall, the structural parameters of compound **1** are comparable to those previously reported by us for the symmetrical dinuclear Ru complexes of type **a** and **b** [3].

**Preparation of mixed-valence monoanionic Ru(III)/Ru(II) dinuclear compounds.**

With a synthetic procedure similar to that described for **1** and **2**, we prepared also mixed-valence monoanionic Ru(III)/Ru(II) dinuclear compounds of formula $[NH_4]$[{*trans*-$RuCl_4(Me_2SO$-*S*)}(μ-L){*cis,cis,cis*-$RuCl_2(Me_2SO$-*S*$)_2$(CO)}] (L = pyz (**3**) and pym (**4**)); the bridging ligand L was first mono-coordinated to the Ru(II) precursor *cis,fac*-$RuCl_2(Me_2SO)_3(CO)$ and the resulting neutral complex *cis,cis,cis*-$RuCl_2(Me_2SO$-*S*$)_2$(CO)(L) [7] was treated with $[NH_4]$[*trans*-$RuCl_4(Me_2SO$-*S*$)_2$] in a chloroform/methanol mixture (Scheme 2).

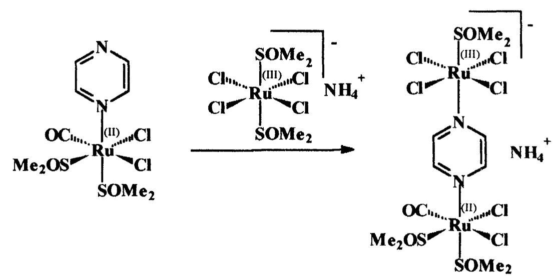

Scheme 2. Reactivity pathway leading to mixed-valence monoanionic Ru(III)/Ru(II) dinuclear compound $[NH_4]$[{*trans*-$RuCl_4(Me_2SO$-*S*)}(μ-pyz){*cis,cis,cis*-$RuCl_2(Me_2SO$-*S*$)_2$(CO)}] (**3**).

Complexes **3** and **4** are fairly soluble in aqueous solution. The $D_2O$ $^1H$ NMR spectrum of complex **3** is characterized by a broad resonance at ca. δ = –14, attributed to the sulfoxide S-bonded to the Ru(III) unit, and by four sharper resonances between δ = 2.13 and 2.82, attributed to the four diastereotopic methyls of the two inequivalent sulfoxides S-bonded to the Ru(II) fragment; with respect to the normal region for $Me_2SO$-*S* coordinated to Ru(II) (δ = 3.3 ÷ 3.6), these resonances are upfield shifted of about 1 ppm by the nearby paramagnetic Ru(III) nucleus. Applying an inversion recovery pulse sequence (π-τ-π/2) we found that all four Ru(II)–$Me_2SO$-*S* resonances are positive for τ = 100 ms, while the two more downfield signals become negative for τ = 50 ms (Figure 4); such resonances were thus attributed to the $Me_2SO$-*S* trans to pyz, whose protons are expected to have longer $T_1$ relaxation times as they are the furthest from the paramagnetic Ru(III) center.

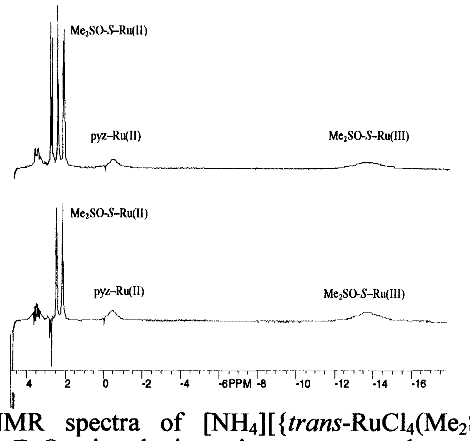

Figure 4. $^1H$ NMR spectra of $[NH_4]$[{*trans*-$RuCl_4(Me_2SO$-*S*)}(μ-pyz){*cis,cis,cis*-$RuCl_2(Me_2SO$-*S*$)_2$(CO)}] (**3**) recorded in $D_2O$ using the inversion-recovery pulse sequence with τ = 100 ms (top) and τ = 50 ms (bottom).

Finally, a broad and relatively weak peak at δ = -0.4 was attributed to the two pyz protons closest to the Ru(II) necleus, while the resonance of the other two protons is broadened beyond detection by the nearby Ru(III) center.

**Chemical behavior in physiological solution.**

Aqueous solutions of the dimeric species **1-4** are characterized by a rather strong absorption band (ε 5000 ÷ 9000 $dm^3$ $mol^{-1}$ $cm^{-1}$) in the visible region of the electronic spectrum (380÷400 nm), attributed to a charge-transfer from the chloride ligands to Ru(III). The time profiles of the visible spectra were used to investigate the chemical behavior of **1-4** at physiological pH.

The spectral changes of the symmetrical dinuclear species **a** at pH = 7.4 were relatively simple and attributed to stepwise chloride hydrolysis from both Ru(III) centers [3]; conversely, for **1** and **2** a general decrease of the main absorption band, not accompanied by the formation of any new clear spectral feature, was observed. When the chemical behavior of **1** was monitored by $^1$H NMR spectroscopy, the resonance of free dimethylsulfoxide at δ = 2.72 increased with time; dissociation of the O-bonded ligand prevailed, as its resonance decreased faster than that of $Me_2SO$-*S*. Moreover, even though no relevant fragmentation of the dinuclear structure was observed, relatively sharp resonances increased with time at δ = 2.55 and 1.90, which might be attributed to $Me_2SO$ coordinated through O and through S, respectively, to a Ru(II) center in a mixed-charge dinuclear species; thus, partial self-reduction of the $RuCl_3(Me_2SO)_2$ half of the dimeric compound is likely to occur. A similar self-reduction process has been already hypothesized for explaining the chemical behavior of the mononuclear species $RuCl_3(Me_2SO)_3$ in aqueous solution [6]. Overall, the spectral behaviors of **1** and **2** were attributed to multiple and contemporaneous hydrolytic processes concerning chloride and dimethylsulfoxide ligands, and occurring with relatively slow rates (hours), accompanied by partial self-reduction of the neutral $RuCl_3(Me_2SO)_2$ fragment.

The spectral changes observed for **3** and **4** were more straightforward and qualitatively very similar to those observed for NAMI-A under the same conditions and were attributed to stepwise chloride hydrolysis from the Ru(III) fragment only. Figure 5 reports the spectral variations for **4**; in the first step, which is complete in ca. 90 min at 25.0 °C, the main absorption band at 400 nm is gradually replaced by a new band at 350 nm, typical of a *mer*-$RuCl_3$ unit, which was thus attributed to the neutral [{*mer*-$RuCl_3(H_2O)(Me_2SO$-*S*)}(μ-L){*cis,cis,cis*-$RuCl_2(Me_2SO$-*S*$)_2$(CO)}] species. The clean isosbestic point at 375 nm in the visible spectra, supported by time-driven $^1$H NMR spectra under the same conditions, indicates that no relevant fragmentation of the dinuclear structure occurs during this step. The first hydrolysis is followed by a slower step that involves a decrease of the 350 nm band and was attributed to the hydrolysis of a second chloride from the Ru(III) fragment.

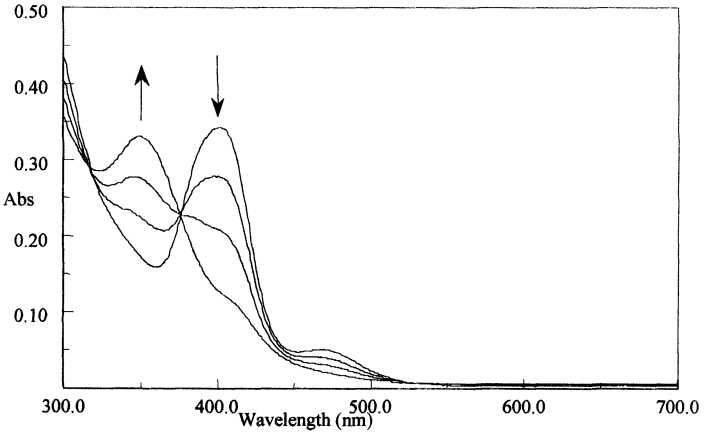

Figure 5. Spectral changes in the visible region during hydrolysis of the first chloride from [$NH_4$][{*trans*-$RuCl_4(Me_2SO$-*S*)}(μ-pym){*cis,cis,cis*-$RuCl_2(Me_2SO$-*S*$)_2$(CO)}] (**4**) (25.0 °C, 0.1 mol $dm^{-3}$ phosphate buffer, pH 7.4, 0.9% NaCl, [**4**] = $10^{-4}$ mol $dm^{-3}$, scan time interval = 30 min).

**Effect of biological reducing agents.**

As in the case of the corresponding dianionic and neutral dinuclear species, [{*trans*-$RuCl_4(Me_2SO$-*S*)$\}_2$(μ-L)$]^{2-}$ (**a**) and [{*mer*-$RuCl_3(Me_2SO$-*S*)($Me_2SO$-*O*)$\}_2$(μ-L)] (**b**), we found that addition of one equivalent of ascorbic acid to a physiological solution of either **1** or **2** led to the immediate and complete reduction to Ru(II) species, as indicated by NMR evidence. The case of **1** will be described in more detail. Comparison with the $^1H$ NMR spectra of the reduced forms of the dinuclear and mononuclear reference compounds $[\{trans\text{-}RuCl_4(Me_2SO\text{-}S)\}_2(\mu\text{-}pyz)]^{2-}$, $[\{mer\text{-}RuCl_3(Me_2SO\text{-}S)(Me_2SO\text{-}O)\}_2(\mu\text{-}pyz)]$, $[trans\text{-}RuCl_4(Me_2SO\text{-}S)(pyz)]^-$, $[mer\text{-}RuCl_3(Me_2SO\text{-}S)(Me_2SO\text{-}O)(pyz)]$, $[trans\text{-}RuCl_4(Me_2SO\text{-}S)_2]^-$, and $[mer\text{-}RuCl_3(Me_2SO\text{-}S)_2(Me_2SO\text{-}O)]$, allowed us to assign most of the resonances of the most abundant species according to the following general rules: 1) reduction involves *O* to *S* linkage isomerization of the equatorial $Me_2SO$-*O* ligand accompanied by its partial dissociation (ca. 25%); 2) chloride hydrolysis from the *mer*-$RuCl_3(Me_2SO\text{-}S)$ fragment occurs selectively in the position trans to $Me_2SO$-*S*; 3) resonances of $Me_2SO$-*S* methyl groups occur at $\delta = 3.6 \div 3.7$ when the sulfoxide is bound to $[\{mer\text{-}RuCl_3(Me_2SO\text{-}S)(H_2O)\}(1/2\mu\text{-}pyz)]$ unit and at $\delta = 3.3 \div 3.5$ when bound to $[\{mer\text{-}RuCl_3(Me_2SO\text{-}S)_2\}(1/2\mu\text{-}pyz)]$ unit; 4) the resonances of pyrazine are indicative of its mode of coordination: terminal (multiplets at $\delta = 9.2 \div 9.5$ (H2,6) and at $\delta = 8.6 \div 8.9$ (H3,5)) or bridging ($\delta = 9.3 \div 9.8$); 5) protons of bridging pyz resonate as a singlet when the two Ru(II) units are equal to one another and as two equally intense multiplets when they are different.

The spectrum of **1** taken immediately after reduction is quite complicated; the region of pyrazine resonances (Figure 6) indicates the presence of at least four non-symmetrical and of one symmetrical (singlet at $\delta = 9.65$) dinuclear species. The spectrum, however, is in rapid evolution and after 15 min it becomes considerably simpler, with two main species, one symmetrical (**1N**) and the other non-symmetrical (**1C**) , in a ca. 1:3 ratio (Figure 6).

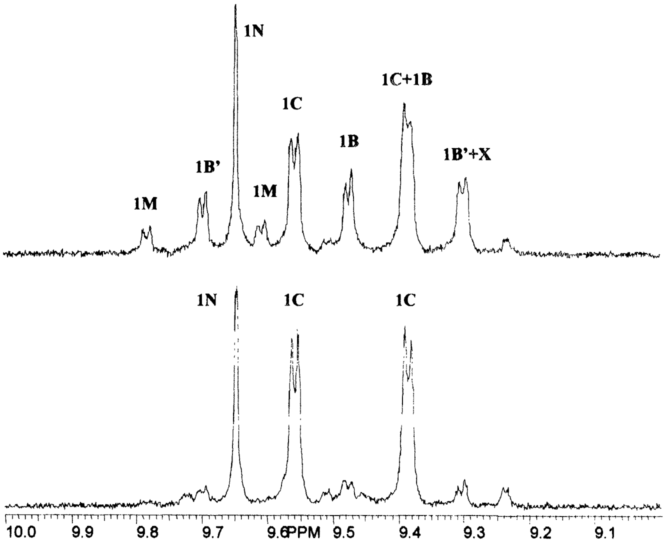

Figure 6. Region of the pyrazine resonances in the $^1H$ NMR spectrum of $[NH_4][\{trans\text{-}RuCl_4(Me_2SO\text{-}S)\}(\mu\text{-}pyz)\{mer\text{-}RuCl_3(Me_2SO\text{-}S)(Me_2SO\text{-}O)\}]$ (**1**) taken immediately after reduction with ascorbic acid (top) and after 15 min (bottom) ($D_2O$, phosphate buffer pH* 7.4). See Scheme 3 for labeling scheme. X indicates a mononuclear species.

The overall chemical behavior of **1** after reduction is summarized in Scheme 3. Both the trianionic species **1A** and **1M** obtained upon reduction of **1** (**1M** derives from dissociation of the equatorial $Me_2SO$ ligand) hydrolyse quite rapidly (minutes) one chloride. Chloride hydrolysis occurs with comparable rates from the $RuCl_4$ and from the *mer*-$RuCl_3(Me_2SO\text{-}S)$ fragments, thus the time-profiles of the transient species **1B** and **1B'** are similar; hydrolysis from the *mer*-$RuCl_3(H_2O)$ fragment is much slower. Species **1C** and **1N** are by far the most abundant between 15 and 60 min after reduction; for longer observation times, formation of species **1D** and **1O**, derived by further hydrolysis of a chloride from **1C** and **1N**, respectively, occurs (the geometry, either *cis* or *trans* of the $Ru(H_2O)_2$ fragment in **1D** and **1O** was not determined). Moreover, the resonances of mononuclear species, of free pyrazine and dimethylsulfoxide become increasingly relevant. Formation of all possible mononuclear species (either bearing or not terminally coordinated pyz) in comparable amounts indicates that fracture of both Ru–pyz bonds is equally likely.

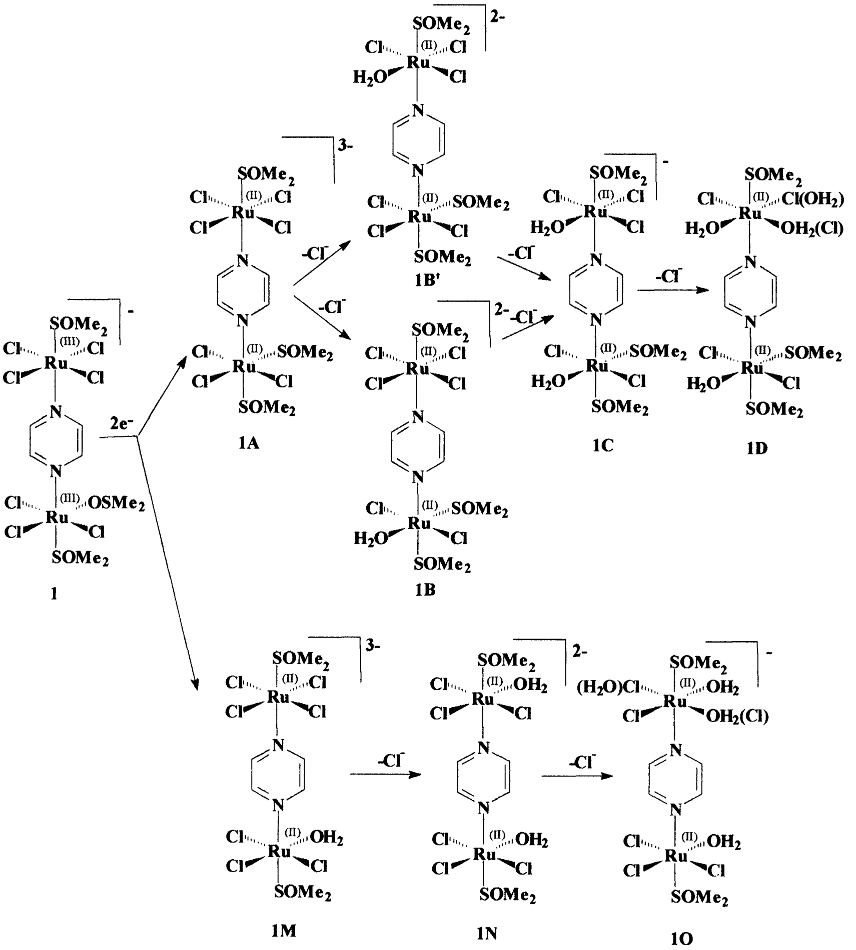

Scheme 3. Main species found in physiological solution within 2h after reduction of $[NH_4]$[{*trans*-$RuCl_4(Me_2SO\text{-}S)$}(μ-pyz){*mer*-$RuCl_3(Me_2SO\text{-}S)(Me_2SO\text{-}O)$}] (**1**) by addition of one equivalent of ascorbic acid (25 °C).

The behavior of the pyrimidine compound **2** after reduction is qualitatively very similar to that of **1**, except that the chloride hydrolytic processes are ca. 20% faster and also the formation of monomeric species is more pronounced.

In both cases, the reduction of the dimeric compounds is also accompanied by a remarkable increase in solubility due to the increased charge and by a dramatic change in color, from orange to dark red (the main absorption band at 380 nm is replaced by an intense and broad band at 447 nm, attributable to a $t_{2g} \rightarrow \pi^*$ charge-transfer absorption as in the case of the symmetrical dimeric species [3]).

Treatment. of physiological solutions of the mixed-valence dinuclear species [{*trans*-$RuCl_4(Me_2SO\text{-}S)$}(μ-L){*cis,cis,cis*-$RuCl_2(Me_2SO\text{-}S)_2(CO)$}]$^-$ (L = pyz (**3**) and pym (**4**)) with one equivalent of ascorbic acid led similarly to the complete and rapid reduction of the Ru(III) fragment. Also in this case the chemical behavior of the reduced species was investigated mainly by $^1H$ NMR spectroscopy. The behavior of **3** after reduction is summarized in Scheme 4 and is substantially similar to that of the corresponding monomeric species [*trans*-$RuCl_4(Me_2SO\text{-}S)(pyz)$]$^-$ [3] because the *cis,cis,cis*-$RuCl_2(Me_2SO\text{-}S)_2(CO)$ fragment is inert and all hydrolytic processes occur on the other half of the dimer. Also fracture of the Ru(II)–pyz bond occurs exclusively at the site of the reduced Ru atom, generating the mononuclear species *cis,cis,cis*-$RuCl_2(Me_2SO\text{-}S)_2(CO)(pyz)$ (identified by comparison with the resonances of a pure sample).

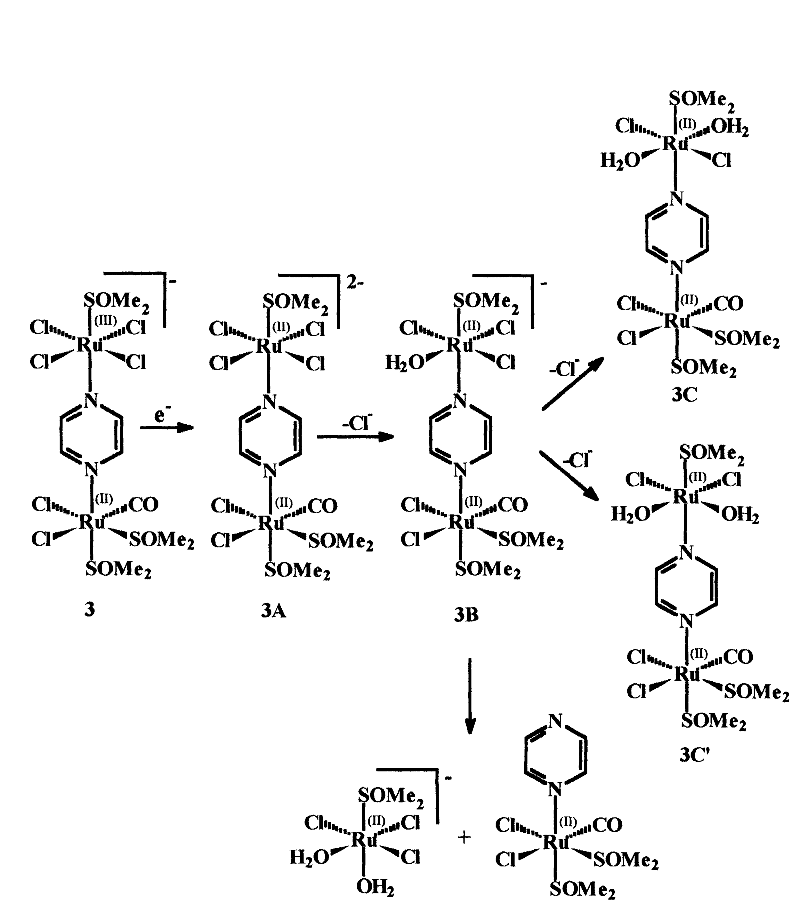

Scheme 4. Main species found in physiological solution within 2h after reduction of $[NH_4][\{$*trans*-$RuCl_4(Me_2SO$-*S*$)\}(\mu$-pyz$)\{$*cis,cis,cis*-$RuCl_2(Me_2SO$-*S*$)_2(CO)\}]$ (**3**) by addition of one equivalent of ascorbic acid (25 °C).

In more details, the dianionic species **3A** disappears within 20 min after reduction, generating the monoanion $[\{$*mer*-$RuCl_3(Me_2SO$-*S*$)(H_2O)\}(\mu$-pyz$)\{$*cis,cis,cis*-$RuCl_2(Me_2SO$-*S*$)_2(CO)\}]^-$ (**3B**), which is the prevailing species in the first hour after reduction. **3B** either hydrolyzes a further chloride to give the *trans* and *cis* geometric isomers **3C** and **3C'** in a ca. 1:2 ratio (in this case the pyz resonances of the two isomers are distinguishable, but assignment to each isomer was not possible) or splits into mononuclear species which represent 20% of the total after 2.5 h. The pyrimidine dinuclear species **4** behaves similarly to **3** upon reduction, except that the hydrolytic processes are more rapid and the splitting of the Ru-N bond, which yields selectively *cis,cis,cis*-$RuCl_2(Me_2SO$-*S*$)_2(CO)$(pym), occurs to a larger extent (50% of mononuclear species 3h after reduction). The reduction of **3** and **4** involves an increase in solubility and a change of color similar to that observed upon reduction of the Ru(III)/Ru(III) dimeric species **1** and **2**.

In conclusion, the chemical behavior of the unsymmetrical Ru(III)/Ru(III) species $[\{$*trans*-$RuCl_4(Me_2SO$-*S*$)\}(\mu$-L$)\{$*mer*-$RuCl_3(Me_2SO$-*S*$)(Me_2SO$-*O*$)\}]^-$ (L = pyz (**1**), pym (**2**)) after treatment with biological reducing agents in physiological solution resembles that of the corresponding dianionic and neutral dinuclear species, $[\{$*trans*-$RuCl_4(Me_2SO$-*S*$)\}_2(\mu$-L$)]^{2-}$ and $[\{$*mer*-$RuCl_3(Me_2SO$-*S*$)(Me_2SO$-*O*$)\}_2(\mu$-L$)]$, respectively. As reported in Scheme 3 (and not considering fragmentation) reduced **1** and **2**, after chloride and/or sulfoxide hydrolysis, are bi-functional binders at one site and mono-functional binders at the other (coordinated water is a labile ligand); thus, their adducts with biological targets are likely to be different from those obtained upon coordination of the corresponding mononuclear species. On the other hand, the mixed-valence dinuclear compounds $[\{$*trans*-$RuCl_4(Me_2SO$-*S*$)\}(\mu$-L$)\{$*cis,cis,cis*-$RuCl_2(Me_2SO$-*S*$)_2(CO)\}]^-$ (L = pyz (**3**) and pym (**4**)), owing to the great inertness of the *cis,cis,cis*-$RuCl_2(Me_2SO$-*S*$)_2(CO)(1/2\mu$-L$)$ fragment, both before and after reduction behave substantially like the corresponding mononuclear species [*trans*-$RuCl_4(Me_2SO$-*S*$)(L)]^-$ in which the terminally bonded L ligand can be considered as bearing a bulky substituent on the other N atom.

**ACKNOWLEDGMENTS**
This work was supported by Ministero dell'Università e della Ricerca Scientifica e Tecnologica in the frame of the Project "Pharmacological and Diagnostic Properties of Metal Complexes" (coordinator Prof. G. Natile), and by EU COST Action D8 (Working Group D8/97/0017). We thank Johnson Matthey for a generous loan of hydrathed $RuCl_3$.

**REFERENCES**

1. G. Sava, E. Alessio, A. Bergamo, G. Mestroni, *Topics in Biological Inorganic Chemistry*, Volume 1 "*Metallo-pharmaceuticals*", M. J. Clarke and P. J. Sadler eds., Springer, Berlin, (1999), pp. 143-169.
2. a) N. Farrell, Y. Qu, U. Bierbach, M. Valsecchi, E. Menta, in *Cisplatin - Chemistry and Biochemistry of a Leading Anticancer Drug*, ed. B. Lippert, VHCA (Zurich) and Wiley-VCH (Weinheim), 1999, pp 479-496; b) N. Farrell, *Comments Inorg. Chem.* (1995), **16**, 373; c) Y. Qu, S. G. da Almeida and N. Farrell, *Inorg. Chim. Acta* (1992), **201**, 123.
3. E. Iengo, G. Mestroni, S. Geremia, M. Calligaris, E. Alessio, *J. Chem. Soc., Dalton Trans.* (1999), 3361.
4. E. Alessio, E. Iengo, S. Zorzet, A. Bergamo, M. Coluccia, A. Boccarelli, G. Sava, *J. Inorg. Biochem.* (2000), **79**, 157.
5. E. Alessio, G. Balducci, A. Lutman, G. Mestroni, M. Calligaris, W. M. Attia, *Inorg. Chim. Acta* (1993), **203**, 205.
6. E. Alessio, G. Balducci, M. Calligaris, G. Costa, W.M. Attia, G. Mestroni, *Inorg. Chem.* (1991), **30**, 609.
7. E. Alessio, B. Milani, M. Bolle, G. Mestroni, P. Faleschini, F. Todone, S. Geremia, M. Calligaris, *Inorg. Chem.* (1995), **34**, 4722.
8. G. M. Sheldrick, *Acta Cryst.* (1990), **A46**, 467.
9. G. M. Sheldrick, SHELXL-97, *Program for crystal structure refinement*, University of Göttingen, Germany, 1997.
10. L. J. Farrugia, *J. Appl. Cryst.* (1999), **32**, 837.
11. M. Stener, M. Calligaris, *J. Mol. Struct. (Theochem.)* (2000), **497**, 91.
12. M. Calligaris, O. Carugo, *Coord. Chem. Rev.* (1996), **153**, 83.
13. M. Calligaris, *Croat. Chem. Acta* (1999), **72**, 147.

